# The Effect of Hypnotherapy on the Resilience of Women With Polycystic Ovarian Syndrome: Study Protocol for a Randomized Controlled Trial

**DOI:** 10.1002/hsr2.71608

**Published:** 2025-12-09

**Authors:** Soghra Khani, Zohreh Shahhosseini, Mahdi pourasghar, Roya Nikbakht, Mahtab Hajimohammadreza

**Affiliations:** ^1^ Department of Reproductive Health and Midwifery, Sexual and Reproductive Health Research Center Mazandaran University of Medical Sciences Sari Iran; ^2^ Department of Psychiatry, Psychiatry and Behavioral Sciences Research Center, Addiction Institute Mazandaran University of Medical Sciences Sari Iran; ^3^ Department of Biostatistics and Epidemiology, Faculty of Health Mazandaran University of Medical Science Sari Iran; ^4^ Midwifery Counseling Mazandaran University of Medical Sciences Sari Iran

**Keywords:** hypnotherapy, polycystic ovarian syndrome, quality of life, resilience

## Abstract

**Background and Aims:**

Polycystic ovarian syndrome, with a mean incidence of 5–10, is the most common endocrine disorder affecting women of reproductive age. These patients suffer from a wide range of symptoms that are inversely related to their resilience and quality of life. Thus, this study protocol was prepared to determine the effect of hypnotherapy intervention on the resilience of patients with polycystic ovary syndrome.

**Methods:**

The current study protocol is a randomized clinical trial. In total, 56 women diagnosed with polycystic ovarian syndrome will be invited to participate in a 6‐week intervention. After allocating the samples to two control and intervention groups using a block randomized design, for each subject in the intervention group, 6 weekly sessions of hypnotherapy will be held for 40–60 min, and for the control group, routine care will be provided. In this study, the level of patient resilience will be measured as the primary outcome using the Connor‐Davidson Resilience Scale (CD‐RIS). Analysis will be conducted using SPSS V.26. To compare variables between intervention and control groups according to the qualitatively and quantitatively variable nature, the *χ* ^2^ test (if the default is not established, Fisher's exact test) and *t*‐test of two independent samples, analysis of variance, or its non‐parametric equivalent (Mann–Whitney and Kruskal–Wallis) will be used, respectively.

**Discussion:**

This plan focuses on the resilience and quality of life in women with polycystic ovarian syndrome. Considering that hypnotherapy is safe and relatively inexpensive, and has almost no side effects, it is very important to determine whether women with polycystic ovary syndrome could benefit from this psychological technique.

**Trial Registration:**

Iranian Clinical Trial Register IRCT20161126031117N12; Registered V1.0 on May 4, 2022.

## 1 Background

Polycystic Ovarian Syndrome (PCOS), also known as Stein–Leventhal syndrome, is the most common endocrine disorder affecting women of reproductive age, with a mean incidence of 5%–10% [1, 2]. Women with PCOS display various symptoms, including amenorrhea, oligomenorrhea, hirsutism, subfertility or infertility, anovulation, weight gain or obesity, acne vulgaris, and androgenic alopecia [3]. As per the updated Rotterdam Criteria established by the European Society of Human Reproduction and Embryology, along with the American Society for Reproductive Medicine, a diagnosis of PCOS requires meeting at least two out of the three criteria [2, 3]. These criteria include anovulation, excess androgen levels, and polycystic ovarian appearance observed through ultrasound evaluation [4, 5, 6].

The prevalence of PCOS is relatively high in Iran (11.4%) [7]. During the reproductive years, the primary issue faced by the patient is infertility along with irregular ovulation [8]. This syndrome is the leading reason for roughly 75% of infertility resulting from an absence of ovulation [9]. Individuals with PCOS encounter higher risks of endometrial and ovarian cancer [10], delayed menopause [11], type 2 diabetes, hypertension, lipid abnormalities, cardiovascular issues [12, 13], depressive and anxious states [14], social anxiety [15], eating disorders [16], suicide attempts [17], and bipolar disorder [18]. According to the previous studies, women with higher resilience face a lower risk of irregular menstruation. Resilience could be described as the capability to bounce back from stress, maintaining consistent levels of mental and physical performance despite stressors, or as the enhancement of health and adaptability, irrespective of the stress levels individuals encounter [19]. Normal women have higher endurance compared to women with ovarian cysts [20]. In addition, studies indicate that the consequences and complications associated with PCOS cause psychological damage and a notable decline in both quality of life and health‐related quality of life for women affected by this condition [1, 21]. Therefore, the quality of life for women with PCOS has been noted to be lower than that of women without the condition [22] and even inferior to individuals with certain illnesses like diabetes, asthma, and epilepsy [23]. Furthermore, these individuals have a significant risk of experiencing depression and anxiety disorders [24].

So, PCOS is a long‐term condition that impacts both the physical and mental well‐being of those affected, significantly diminishing their quality of life [25]. Currently, various methods including pharmacological methods such as Metformin [26], Letrozole and Clomiphene Citrate [27], Finasteride [28], etc. as well as nonpharmacological methods such as cognitive‐behavioral therapy [29, 30], acupuncture [31], lifestyle management [32, 33], relaxation [34], acceptance and commitment therapy [35], yoga [36], etc. are used to reduce the complications caused by PCOS. One of the nonpharmacological methods used in gynecological diseases is hypnotherapy [37, 38]. Hypnosis is a normal occurrence that nearly everyone goes through occasionally. Hypnotic trance states resemble daydreaming and rely on the notion that many individuals are somewhat suggestible [39]. Hypnotherapy is a treatment that employs a hypnotic approach to access an individual's subconscious mind. Hypnotherapy in surgery can provide a comfortable and relaxing experience for patients. Hypnotherapy, in principle, can evoke sensations of joy, contentment, security, and ease, enabling an individual to feel empowered to combat or lessen their anxiety levels [40]. Hypnosis is secure and fairly affordable, can be learned by many determined individuals to some extent, and has almost no side effects [39]. According to the extensive search that has been done in the available databases, so far, no clinical trial study has been conducted about the impact of hypnosis on the psychological complications of polycystic ovary syndrome in Iran and international studies. For this reason, we tried to design a study to investigate the impact of hypnotherapy treatment on the resilience of individuals with polycystic ovary syndrome.

## 2 Methods

### 2.1 Objectives

The primary objective of this study is to assess the impact of hypnotherapy on the resilience of individuals suffering from polycystic ovary syndrome (PCOS). The particular aims of this study are to assess and contrast the levels of resilience, quality of life, anxiety, and depression in both the control and intervention groups before the intervention, right after the intervention, and 4 weeks post‐intervention.

### 2.2 Study Design

This study is a randomized clinical trial consisting of two parallel groups of intervention and control.

### 2.3 Study Registration

The researcher received their code after registering the project in the Ethics Committee of Mazandaran University of Medical Sciences (IR.MAZUMS.REC.1400.11670) and registering it in the Clinical Trial Registration Center of Iran (IRCT20161126031117N12).

### 2.4 Study Setting and Participants

In this study, the research community is women with polycystic ovarian syndrome, and the research setting is healthcare centers and gynecological and midwifery offices from different parts of Tehran, Iran. These settings are usually staffed by healthcare providers, gynecologists, and midwives to whom different patients with different socioeconomic statuses and educational backgrounds would refer. Recruitment will occur through advertisements placed in waiting rooms in healthcare centers, gynecological and midwifery offices in Tehran, as well as media outlets.

#### 2.4.1 Inclusion and Exclusion Criteria

The inclusion criteria will include 19–49‐year‐old Iranian women with confirmed polycystic ovarian syndrome according to Rotterdam criteria who have attended to visit for the first time because of this disease, not being infertile, and undergoing assisted reproductive therapy, literacy in reading and writing in Persian, and being satisfied to participate in the research. In addition, exclusion criteria will be an occurrence of any kind of acute complication affecting the quality of life and resilience during the intervention, (such as the death of important people in a person's life) [41], being suffering from other endocrine diseases or any other chronic diseases which affect patient's quality of life; for instance, chronic debilitating liver and kidney diseases [41], being psychotic and having serious thoughts of harm to oneself and others (recognized by a psychiatrist) [42], history of seizures [42], using psychotropic drugs and any kind of drug abuse [30, 41] and participating in similar training sessions [41].

### 2.5 Sample Size and Sampling Method

#### 2.5.1 Sample Size

According to Fathi et al.'s study [41], the average resilience in the control group was 48.17 ± 20.61, and in the intervention group was 69.80 ± 23.38. By applying these findings and taking into account a type 1 error of 0.05 and a test power of 0.90, the sample size for every group was calculated using the following formula:

Therefore, 22 people in each group and a total of 44 people will enter the study. But considering that in this study, resilience is measured in three stages (before the intervention, right after the intervention, and 4 weeks post‐intervention). The samples may have dropped. For this problem, the final sample volume was determined with a 20% drop:

The total sample size was 55, considering the 20% attrition, and as regards, we have two groups, so we considered the total sample size to be 56 people. Therefore, 28 samples are included in the study in each group (intervention and control).

#### 2.5.2 Sampling

The sampling method will be done in a convenience method. Following the receipt of authorization from the Ethics Committee of Mazandaran University of Medical Sciences, the researcher registered the project in the Iranian Clinical Trial Registration Center, received its code, attended and presented a letter of introduction, and went to midwifery and gynecology offices in Tehran. In the first stage, the call will be announced through the offices; qualified people will be invited to take part in the study if they wish. Then, a list of qualified people will be prepared, and all the people who are eligible and have declared their preparation will be listed with a code (instead of specifications) in an Excel sheet.

### 2.6 Random Allocation

In this study, the random blocking method will be utilized for the random allocation of samples in the intervention and control groups, based on marital status (single and married) and BMI (< 20, 20–25, and > 25). For this purpose, double blocks will be considered. The two possible blocks are TC and CT, where T stands for the intervention group, and C stands for the control group. Numbers will be generated randomly (with the RANDBETWEEN command in Excel software and ranges 1 and 2), and according to the production values of one of the blocks (for production number 1 CT block, for production number 2 TC blocks) will be selected and samples will be allocated. For instance, two married women with a body mass index of 20–25 have been referred, one assigned to the intervention group and the other to the control group. In Excel software, between numbers one and two, assuming the number two is selected randomly. Therefore, these two people are assigned to the TC block. Accordingly, these two people are assigned to refer to this block in order. The first person enters the intervention group, and the second person enters the control group. Now, if the number one is chosen, these two people will be assigned to the CT block [43].

### 2.7 Blinding

**Figure 1:**
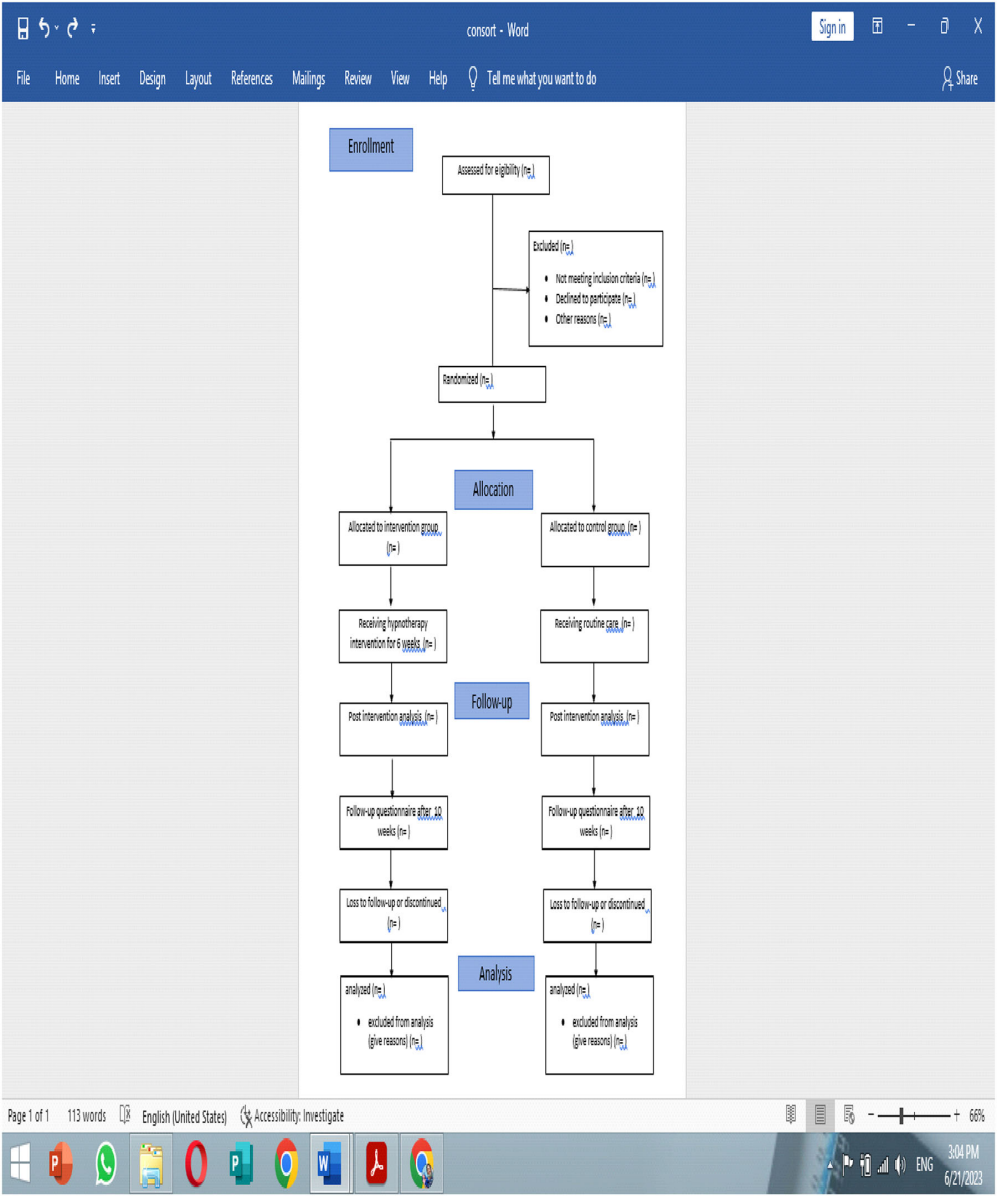
Consolidated standards of reporting trials flow diagram estimating patient screening, enrollment, and response rate.

It will be impossible to keep participants blind during the intervention process, as a result of the nature of the intervention [44]. Blinding will be done at the assessor level [45]. In this way, after concluding the work, the research colleague hands over the questionnaires to the samples, and after completing the questionnaire, a box will be placed there, and the participants will put the completed questionnaire in the box. Groups are named A and B. The statistical analyst is also blinded, and at the end of the analysis, the coded groups will be identified [44, 45, 46]. Figure 1 is the CONSORT flow diagram and includes the calculation of the numbers of eligible, screened, enrolled, and analyzed participants.

### 2.8 Adverse Events

This is a low‐risk study; we do not prognosticate any adverse events related to the study intervention (hypnosis). However, the Principal Investigator and the study team will evaluate and promptly address any adverse events. Furthermore, other anticipated issues or events will be evaluated within 72 h, and serious adverse events will be assessed.

### 2.9 Equipment

#### 2.9.1 Data Collection Tools

In this study, the level of patient resilience will be measured as the primary outcome using the Connor‐Davidson Resilience Scale (CD‐RIS). The reliability and validity of the Persian Resilience Scale have been investigated and validated in preliminary studies in patient and normal samples. Mohammadi used Cronbach's alpha coefficient; the reliability scale reached 0.89, and the worth of each item alongside the overall score using the correlation coefficient ranged from 0.41 to 0.64 [47]. Samani and Sahragard, in a study on students in Shiraz, achieved scale reliability through Cronbach's alpha coefficient of 0.87 [48].

Additionally, the quality of life of the patients (as the secondary outcome) will be measured using the PCOS Health‐Related Quality of Life Questionnaire (PCOSQ). Amini et al. showed that the Iranian version of PCOSQ has acceptable validity and reliability and is an appropriate tool for measuring the quality of life of patients with polycystic ovary syndrome. To assess the reliability of the questionnaire, they employed the method of evaluating the internal consistency of the instrument. The findings demonstrate that the Cronbach's alpha coefficient for the entire questionnaire was 0.90, for emotional aspects it was 0.80, for hirsutism it was 0.87, for weight it was 0.77, for infertility issues it was 0.84, and for menstrual issues it was 0.52. In addition to testing the validity of the questionnaire, a type of validation, namely item‐level correlation and comparison between known groups, was used. So, each question shows the highest correlation coefficient with its relevancy domain, and all coefficients are greater than 0.4 [49].

Eventually, their anxiety and depression (as the secondary outcome) will be evaluated using the Hospital Anxiety and Depression Scale (HADS). Kaviani et al. showed in their research that the HADS scale and subscales of anxiety and depression have good validity, reliability, and internal consistency with a Cronbach's alpha coefficient of 0.07 for the seven items of the depression scale, and 0.85 for the seven items under the anxiety scale [50].

#### 2.9.2 Data Management

To complete the questionnaires, participants will be given randomly assigned ID numbers. Separate linking lists containing names and ID numbers will be kept confidential on a server that can only be accessed by project members and Systems Administrators (who are trained in conducting ethical research with human participants). Upon completion of the project and verification of the data, the linking list will be destroyed, and the data will become anonymous. The study coordinator, who is not blinded to the randomization, will document recruitment, attrition, and safety. Study data will consistently be kept securely, either in a locked cabinet or on computers with password protection, accessible only to study team members.

### 2.10 Interventions

After allocating the samples to two control and intervention groups, they will be provided with the questionnaires. Then, patients of both groups receive standard treatments related to women with PCOS (including oral contraceptives, spironolactone, and metformin) [30, 51] and will be visited and treated by a gynecologist. Patients will also be recommended to follow a diet of their choice with an intake of 1500–1800 kcal per day [29], as well as a gentle aerobic exercise activity of 150 min per week [52] during the study. In addition, they should not use other relaxation methods and counseling sessions during the research. Also, the participants will be asked not to use cosmetic procedures (including laser and Botox, etc.) during the study, and they will be asked in each session, and if they are used, it will be recorded.

#### 2.10.1 Hypnotherapy Intervention Group

In the intervention group, hypnotherapy is performed by a qualified researcher who is certified by the Iranian Association of Clinical Hypnosis and holds a valid credential. For every participant in the intervention group, there will be 6 weekly hypnotherapy sessions lasting 40–60 min each (Table 1).

**Table 1:** The content of six hypnotherapy sessions.

Sessions	The subject matter of the session
First	Taking a history, fully explaining how to do the work, educating and preparing the patient, eliminating the misunderstanding of hypnosis, and completing the questionnaires (Patients are also asked to describe a place in the real world that gives them a sense of safety and comfort).
Second	Induction and deepening, and direct instincts to increase relaxation and conditioning for rapid hypnosis in the next sessions.
Third	Strengthening the ego to increase self‐esteem and increase the endurance and resilience to life issues in the individual.
Fourth	Age regression and dealing with negative emotions, and then increasing calmness.
Fifth	Age progression and conditioning to improve lifestyle.
Sixth	Reviewing the previous sessions, the mental experience of a favorable physical feeling, and focusing on increasing relaxation in the whole body and mind. Completing the questionnaires again.

#### 2.10.2 Control Group

For the control group, routine care will be done (Routine care means receiving standard treatments by a gynecologist and balanced physical activity and diet).

Questionnaires will be done simultaneously with the intervention group at all stages.

Participants' records will be organized numerically and kept in a secure, easily accessible way and location. Records of participants will be maintained for 2 years following the conclusion of the study.

### 2.11 Protocol Deviation

Deviations or protocol violations may have occurred in this study and will be reported. An instance of deviation in a procedure is a follow‐up appointment occurring at a slightly varied time frame than mandated by the protocol; patients with these deviations will be incorporated into the intention‐to‐treat analysis. All study subjects randomly assigned to the studied groups during randomization processes, regardless of the type of intervention received during the study, their violation of protocol, discontinuation, and withdrawal from participating in the study, as well as all cases that occurred after randomization, will be included in the intention‐to‐treat analysis.

### 2.12 Modification to the Protocol

Any modifications to the procedure that could influence the study's execution, the patient's potential benefit, or the patient's safety, such as alterations in study objectives, design, patient population, sample size, study methods, or important administrative elements, will necessitate official documentation. This modification will be concurred by the research team and sanctioned by the Ethics Committee.

### 2.13 Statistical Analysis

The data from quantitative variables will be described by calculating the mean ± standard deviation, and qualitative variables will be described in terms of frequency and percentage. To compare variables between intervention and control groups according to the qualitative and quantitative variable nature, the *χ* ^2^ test (if the default is not established, Fisher's exact test) and *t*‐test of two independent samples, analysis of variance, or its non‐parametric equivalent (Mann–Whitney and Kruskal–Wallis) will be used, respectively. It should be noted that checking the defaults in terms of normality (Kolmogorov–Smirnov and Shapiro–Wilk test) and equality of variances (Levene and F test) will be on the agenda. Finally, to compare the mean resilience score between the two intervention and control groups during the study period (before the intervention, right after the intervention, and 4 weeks post‐intervention), and also to control for the effect of intervening factors, repeated measures ANOVA or mixed models will be used for variance analysis. For every outcome, the projected effect size along with its 95% confidence interval will be displayed. The significance level of the tests will be 0.05. The analysis will be conducted using SPSS V.26. The reporting of statistical results will adhere to the Statistical Analyses and Methods in the Published Literature (SAMPL) guideline [53].

### 2.14 Testing of the Hypothesis

The main hypothesis of this study involves examining whether hypnotherapy significantly impacts the resilience of women with PCOS. The research is designed to measure changes in resilience levels before the intervention, right after the intervention, and 4 weeks post‐intervention.

### 2.15 Potential Cofounders

At the start of the research, various demographic and medical variables that could create bias will be documented. Additionally, both groups' participants will be required to continue their regular diet and exercise routine throughout the study. To guarantee that participants adhere to the protocol, their food consumption and exercise levels will be evaluated in each session and documented. Eventually, all possible confounders will be statistically examined, and if a significant difference is found between the groups, their impacts on study results will be modified.

### 2.16 Limitations

Our research might encounter various constraints. Since it is a public study, participants are aware they are engaging in an intervention entitled the Effect of Hypnotherapy on the Resilience of Women with PCOS, and bias is probable in the research outcomes. Subsequently, a random assignment of participants can help attain equilibrium in sociodemographic traits. One of the limitations of this study was the relatively short follow‐up period of 4 weeks. This duration is chosen due to the challenges posed by the COVID‐19 pandemic, during which the study will be conducted. Extending the follow‐up period beyond 4 weeks risks a significant loss to follow‐up due to the uncertainty and constraints experienced by participants during the pandemic. However, we acknowledge that a longer follow‐up period would provide more robust insights into the sustainability of the intervention's effects. Future studies are recommended to include longer follow‐up periods to evaluate the long‐term impact and efficacy of hypnotherapy in improving resilience and other psychological outcomes in women with PCOS. Another drawback of this study is the reliance on self‐reporting tools, and the potential for memory mistakes, ambiguity, sociocultural issues, and personal biases influencing the outcomes is unavoidable. The consultative nature of the intervention and the non‐blinding and concealment of the allocation status of the participants from the researcher's point of view will make performance bias possible in the present study. Last but not least, the possibility of a placebo or confounding effect remains in this protocol since participants in the experimental group receive significantly more interaction with the clinical team compared to those in the control group.

## Author Contributions

**Soghra Khani:** writing – review and editing, writing – original draft, supervision, project administration, methodology, and conceptualization. **Zohreh Shahhosseini:** methodology, conceptualization, writing – original draft, writing – review and editing. **Mahdi Pourasghar:** supervision, writing – review and editing. **Roya Nikbakht:** formal analysis and software. **Mahtab Hajimohammadreza:** writing – original draft, writing – review and editing, conceptualization, project administration, methodology, and data curation.

## Ethics Statement

The trial has been approved by the Ethics Committee of Mazandaran University of Medical Sciences (IR.MAZUMS.REC.1400.11670) and prospectively registered with the Clinical Trial Registration Center of Iran on May 4, 2022: (IRCT20161126031117N12).

## Consent

All the moral rights of the participants are preserved in this study. The consent of all the participants and the confidentiality of their information are promised in this intervention.

All participants will be fully informed about their role, and their information will be kept confidential. They will also be asked to complete a written Informed consent form, which emphasizes that their participation is optional and they can withdraw at any time, and include this statement in the ethical approval section of the manuscript. Due to the intervention's nature, this study poses no adverse effects or harm. To the best of our understanding, the research will not lead to any adverse effects. Every 6 months, a report will be sent to the auditor. We will communicate the findings of this study to important stakeholders by presenting at relevant seminars and publishing in peer‐reviewed journals.

Recruitment was originally scheduled to begin in May 2022, but due to a substantial rise in COVID‐19 cases and the center's inability to refer PCOS women, the process was delayed. As a result, in February 2023, the sampling phase commenced, and it is presently ongoing.

## Conflicts of Interest

The authors declare no conflicts of interest.

## Transparency Statement

The corresponding author, Mahtab Hajimohammadreza, affirms that this manuscript is an honest, accurate, and transparent account of the study being reported; that no important aspects of the study have been omitted; and that any discrepancies from the study as planned (and, if relevant, registered) have been explained.

## Supporting information

Supporting File 1.

Supporting File 2.

## Data Availability

Data sharing not applicable to this article as no data sets were generated or analysed during the current study.
