# Supplementary material for: The Effect of Hypnotherapy on the Resilience of Women With Polycystic Ovarian Syndrome: Study Protocol for a Randomized Controlled Trial
Source: Health Sci Rep. 2025 Dec 9;8(12):e71608. doi: 10.1002/hsr2.71608 (PMC12689265; doi:10.1002/hsr2.71608)
Supplement: Supplementary file 2 — Supporting File 2. [file HSR2-8-e71608-s002.docx]

analyzed (n= )

- excluded from analysis (give reasons) (n= )

analyzed (n= )

- excluded from analysis (give reasons) (n= )

Loss to follow-up or discontinued (n= )

Loss to follow-up or discontinued (n= )

Follow-up questionnaire after 10 weeks (n= )

Follow-up questionnaire after 10 weeks (n= )

Post intervention analysis (n= )

Post intervention analysis (n= )

Receiving hypnotherapy intervention for 6 weeks (n= )

Receiving routine care (n= )

Analysis

Follow-up

Allocated to control group (n= )

Allocation

Allocated to intervention group (n= )

Randomized (n= )

Excluded (n= )

- Not meeting inclusion criteria (n= )
- Declined to participate (n= )
- Other reasons (n= )

Enrollment

Assessed for eligibility (n= )
